# JAK Inhibitors for Crohn's Disease: A Systematic Review and Dose–Response Network Meta‐Analysis of Efficacy and Safety

**DOI:** 10.1002/jgh3.70388

**Published:** 2026-03-13

**Authors:** Fares A. Qtaishat, Ahmad AlKayyat, Jehad A. Yasin, Mohammad‐Amer A.Tamimi, Jana Tarawneh, Layan AlDaher, Sarah Abdallah, Mohammad Moayad Ahmad Alghaniem, Abdallah Abunamoos, Adham Musa, Yousef A. Ateiwi, Yousef Taha, Tarek A. Tamimi

**Affiliations:** ^1^ Faculty of Medicine University of Jordan Amman Jordan; ^2^ Faculty of Medicine Medipol University Istanbul Turkey; ^3^ College of Literature, Science, and the Arts University of Michigan Ann Arbor USA; ^4^ Department of Biology The American University in Cairo Cairo Egypt; ^5^ Division of Gastroenterology and Hepatology, Department of Internal Medicine, School of Medicine The University of Jordan Amman Jordan

## Abstract

Crohn's disease has limited effective treatments, and direct comparative data for Janus kinase (JAK) inhibitors are scarce. We conducted a dose‐dependent network meta‐analysis of randomized controlled trials in adults, searching PubMed, Cochrane Library, Scopus, and Web of Science through February 17, 2025. A Bayesian dose–response model (Emax) was implemented with the MBNMA package in R, estimating risk ratios (RRs) with 95% credible intervals, and risk of bias was assessed with RoB 2. Nine trials (*n* = 4838) evaluated upadacitinib, tofacitinib, and filgotinib across multiple doses. Upadacitinib 45 mg once daily achieved the greatest improvement in CDAI remission versus placebo (RR 1.8, 95% CrI 1.1–3.0). SUCRA rankings placed upadacitinib highest for overall clinical remission, CDAI mean change, and CDAI remission. Meta‐regression showed higher doses were associated with higher remission rates (*p* = 0.041). Dose–response parameters indicated upadacitinib had a higher Emax and lower ED50 than comparators, suggesting greater efficacy and potency. Serious adverse events were more frequent with JAK inhibitors versus placebo (RR 1.61, 95% CI 1.15–2.25; *p* = 0.0082), with increases in sepsis, cardiovascular events, and elevated liver enzymes (*p* < 0.05). Adverse event risk differed by agent (*p* = 0.0008); upadacitinib increased overall adverse events versus placebo (RR 1.19, 95% CI 1.07–1.33) and serious infections (RR 5.39, 95% CI 2.98–9.74). JAK inhibitors improve clinical outcomes in Crohn's disease, with upadacitinib showing the most consistent efficacy, but safety risks warrant careful monitoring and head‐to‐head, long‐term studies.

## 1 Introduction

Crohn's disease (CD) is a chronic inflammatory bowel disease (IBD) that affects millions of people around the world. It's an immune‐mediated disorder that causes irritation and swelling in the tissues of the gastrointestinal tract, potentially impacting any part of it [1]. Patients commonly experience symptoms like abdominal pain, ongoing diarrhea, weight loss, fatigue, and malabsorption of nutrients due to the deep involvement of tissue [2]. As is the case with most immune‐mediated diseases, the pathophysiology of CD involves both genetic factors and environmental influences. Essentially, the disease's underlying mechanism includes irregularities in immune responses that lead to inflammation due to imbalanced immune cells and unusual interactions with gut microbiota [3, 4].

Standard, traditional therapy options include corticosteroids, immunomodulators, and specialized biologic medicines against inflammatory pathways, such as tumor necrosis factor (TNF) [5]. However, the current standard therapy options face many challenges and unmet requirements, such as primary non‐response, loss of response with time, and many side effects [6]. Corticosteroids remain effective for inducing remission, but they are unsuitable for long‐term maintenance due to risks of dependency, bone loss, and infections. Immunomodulators such as azathioprine, 6‐mercaptopurine, and methotrexate are used for maintenance therapy but have delayed onset and require close monitoring for hematologic and hepatic toxicity. The introduction of biologic therapies, including anti‐TNF agents (infliximab, adalimumab), anti‐integrin agents (vedolizumab), and anti–IL12/23 agents (ustekinumab), revolutionized disease management; however, these therapies are limited by primary non‐response, secondary loss of response over time, parenteral administration, and immunogenicity [7, 8]. Consequently, alternative therapies have emerged with different mechanisms of action and targets such as Janus Kinase (JAK) inhibitors. JAK inhibitors can be used to treat various autoimmune and inflammatory conditions beyond CD, including rheumatoid arthritis, ulcerative colitis, and atopic dermatitis [9]. They work by targeting intracellular signaling pathways, offering fast relief and reducing the need for steroids [10]. They have, therefore, been found to counter the limitations and drawbacks of standard therapies by blocking the JAK–STAT pathway, which is crucial to cytokine‐mediated immune activation [11]. Several JAK inhibitors—such as tofacitinib, upadacitinib, and filgotinib—are used to treat many diseases, including ulcerative colitis [12]. Subsequently, this study explores their efficacy and safety in treating Crohn's Disease (CD).

Finding the most effective or safest option is challenging due to the insufficiency of studies comparing various Janus Kinase (JAK) inhibitors [13]. Moreover, the choices for treatment are further complicated by the diversity in efficacy and safety profiles due to differences in study populations, dosage regimens, and outcome definitions. To address this gap, this study aims to compare different JAK inhibitors across several dosages regarding safety and efficacy in Crohn's disease using a network meta‐analysis (NMA), especially focusing on the FDA‐approved upadacitinib regimen, to help gastroenterologists make informed, evidence‐based decisions when selecting and optimizing treatment for their patients with Crohn's disease. Furthermore, our study utilizes a model‐based dose–response framework, which evaluates how dose variations affect different outcomes across different pharmacologic agents. Other objectives include the evaluation of subgroup effects and the assessment of long‐term safety outcomes associated with the use of JAK inhibitors.

## 2 Methods

This study was conducted in adherence to the guidelines outlined in the Preferred Reporting Items for Systematic Reviews and Meta‐Analyses (PRISMA) [14] as well as the Cochrane Handbook for Systematic Reviews of Interventions, version 6.3 [15]. Registry of the protocol for this paper has been completed on the International Prospective Register of Systematic Reviews (PROSPERO); register number: CRD420251012689.

### 2.1 Search Strategy

Several digital databases have been used to conduct a comprehensive literature search from conception until February 17, 2025. These databases include, but are not limited to: PubMed, Cochrane Library, Scopus, and Web of Science. The search strategy involved the usage of relevant keywords and Medical Subject Headings (MeSH) terms in order to identify and select studies that evaluate the efficacy and safety of JAK inhibitors for Crohn's disease. These terms included variations of: “randomized controlled trial,” “inflammatory bowel disease,” “Crohn's disease,” in addition to names of specific JAK inhibitors, such as “Tofacitinib,” “Upadacitinib,” and “Filgotinib.” In addition, Boolean operators (AND, OR) were incorporated to optimize retrieval of the relevant studies. Finally, screening of the bibliographies of included studies and the usage of additional sources such as Google Scholar were both incorporated in the search strategy to identify other potentially eligible articles.

### 2.2 Study Selection

Following the retrieval of relevant studies following the search strategy, all records were imported onto Rayyan [16], an online platform used for systematic review screening. Any duplicate records were removed with the use of EndNote. To conduct this process, three independent reviewers screened the titles and abstracts, and any presenting conflicts were resolved by a fourth reviewer. After this process concluded, any study that met the inclusion criteria proceeded to the next phase, full‐text review.

### 2.3 Inclusion Criteria

In order to be included in the full‐text review phase, each study was checked against a number of inclusion criteria. The study must be a randomized controlled trial (RCTs that evaluates JAK Inhibitors (e.g., Upadacitinib, Filgotinib, Tofacitinib) in adult patients with Crohn's disease). In addition, the study must report efficacy outcomes such as clinical remission (Crohn's Disease Activity Index [CDAI] < 150), endoscopic remission, and response rates. Finally, the study must assess safety outcomes, including serious adverse events, infections, venous thromboembolism (VTE), and cardiovascular risks.

### 2.4 Exclusion Criteria

To prevent the inclusion of non‐relevant studies in the full‐text review phase, exclusion criteria were applied. These criteria included non‐original studies (reviews, commentaries, conference abstracts, case reports, and case series), studies on ulcerative colitis or other inflammatory bowel disease that did not include separate data for Crohn's disease, studies published in languages other than English, and studies that are yet to advance beyond the pre‐clinical or animal stage.

### 2.5 Data Extraction

A standardized collaborative Google Sheet was used by five reviewers to independently extract data based on agreed‐upon variables. The extracted variables included study characteristics (first author, year, country, sample size, study design, phase of RCT), participant demographics (age, sex distribution, concomitant medications), intervention details (JAK inhibitor type, dosage, comparator (placebo and/or biologics such as TNF inhibitors, IL‐12/23 inhibitors)), follow‐up duration, efficacy outcomes (clinical remission (CDAI < 150), endoscopic remission, response rates), and safety outcomes (serious adverse events, infections, VTE, cardiovascular risks). Any discrepancies found were then resolved through discussion between the five independent reviewers or by a sixth reviewer.

### 2.6 Quality Assessment

To assess the risk of bias in the included studies, Cochrane Risk of Bias 2 (Rob 2) was used as the tool of choice [17]. Two reviewers evaluated each study's risk of bias based on five domains: randomization process, deviations from intended interventions, missing outcome data, measurement of the outcome, and selection of reported results. Following this evaluation, each study was placed under categories: low risk of bias, some concerns of bias, or high risk of bias. A third reviewer then resolved any discrepancies present.

### 2.7 Statistical Analysis

We conducted a comprehensive meta‐analysis integrating conventional pairwise comparisons, Bayesian network modeling, and dose–response estimation to evaluate the efficacy and safety of JAK inhibitors in Crohn's disease. Conventional meta‐analyses, carried out with “meta” package on R [18], were performed using fixed‐effect (*I* ^2^ < 50%) and random‐effects (*I* ^2^ ≥ 50%) models with inverse variance weighting and the restricted maximum likelihood (REML) estimator for between‐study variance (τ^2^). Heterogeneity was assessed via the Q statistic, Higgins' *I* ^2^, and the Hartung‐Knapp adjustment. Publication bias was explored using Egger's regression method, and funnel plots were visualized to inspect for symmetry. To compare multiple interventions across studies, a Bayesian network meta‐analysis (NMA) was implemented in GeMTC [19], employing a binomial likelihood with a log link and vague priors. Effect size (Risk Ratio; RR) and 95% credible intervals (CrI) were reported, and ranking probabilities for different treatments were calculated. Intervention ranking was processed using the surface under the cumulative ranking (SUCRA) score and visualized using SUCRA plots. Model convergence was assessed using standard MCMC diagnostics over 20 000 inference iterations following a 5000 burn‐in. To explore dose‐related effects, a model‐based network meta‐analysis using an EMAX model was performed utilizing MBNMAdose R package [20], capturing non‐linear dose–response relationships [21]. Detailed dose–response modeling parameters and results are provided in Supporting Information S2 and S3. Visualizations were done using R 4.3.3 and Python 3.80.

## 3 Results

### 3.1 Search Results

**FIGURE 1:**
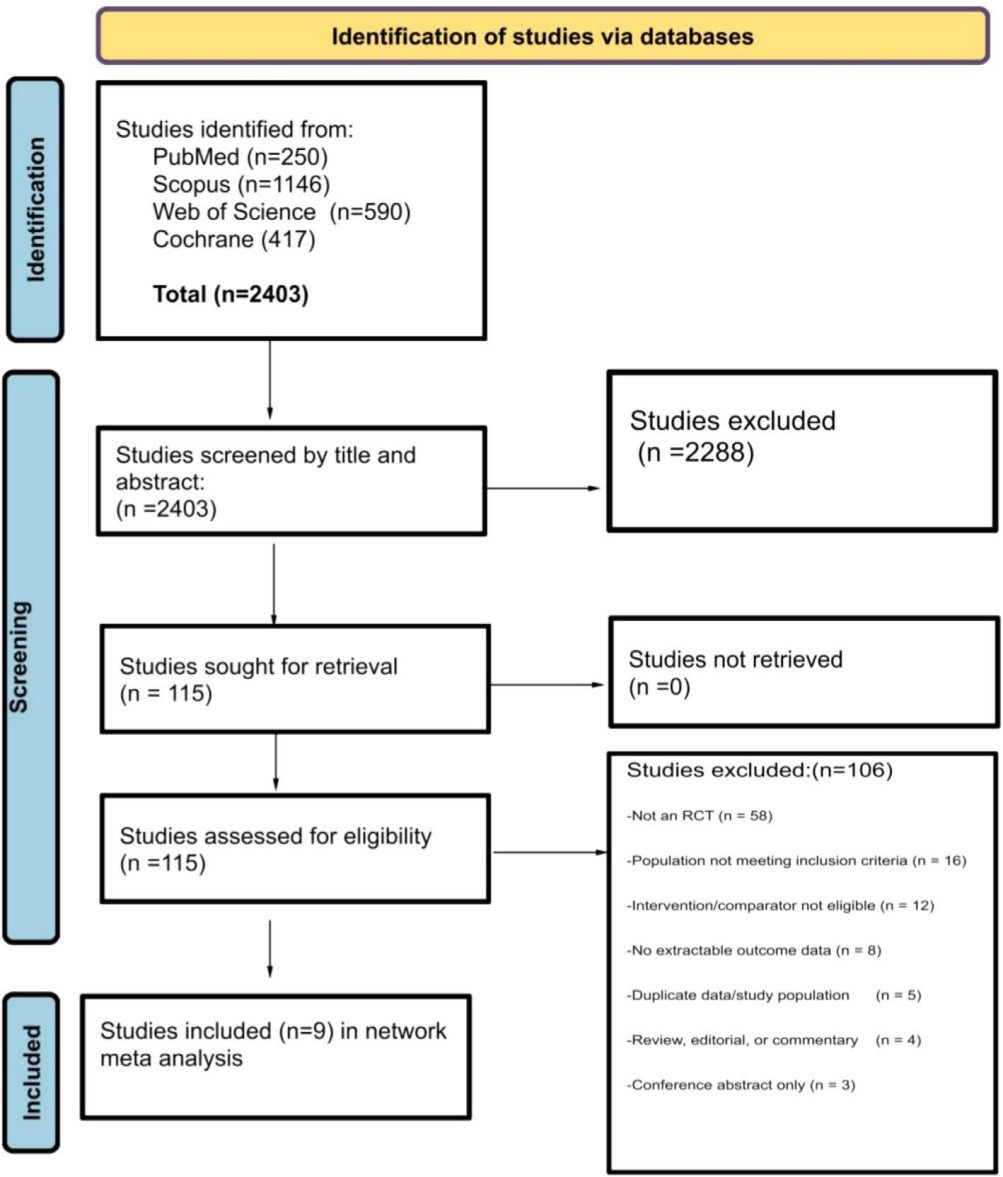
Prisma flow diagram.

Our search retrieved a total of 2403 records that were identified through systematic database searches: 250 from PubMed, 1146 from Scopus, 590 from Web of Science, and 417 from the Cochrane Library. After removal of duplicates and initial screening by title and abstract, 2288 studies were excluded for not meeting the inclusion criteria. The full texts of 115 studies were sought for retrieval, all of which were successfully obtained. Following full‐text review, 106 studies were excluded for the following reasons: not being randomized controlled trials (RCTs) (*n* = 58), population not meeting the inclusion criteria (*n* = 16), ineligible intervention or comparator (*n* = 12), no extractable outcome data (*n* = 8), duplicate data or study population (*n* = 5), publication type being review, editorial, or commentary (*n* = 4), or being a conference abstract only (*n* = 3). Accordingly, 9 RCTs were included in the final network meta‐analysis (Figure 1).

### 3.2 Characteristics of Included Studies

Nine studies comprising 4838 patients with moderate‐to‐severe Crohn's disease were included, several reporting separate induction and maintenance phases. The trials evaluated tofacitinib, filgotinib, and upadacitinib, each compared to placebo across multiple doses. Studies ranged from phase 2 to phase 3, spanning 4 to 58 weeks. Baseline characteristics were well balanced, where mean CDAI scores ranged from 190 to 355. Most patients were aged 37–42 years, with a balanced sex distribution. Prior biologic exposure was frequent. The evidence network was well connected, permitting robust comparisons of all interventions (Table 1).

**TABLE 1:** Baseline study characteristics of placebo‐controlled double‐blinded randomized trials of JAK inhibitors in Crohn's disease.

Study reference	Phase and type	Study duration	Arms	Dose	Number of participants	Age mean (SD)	Female (*n*)	Prior biologic use (*n*)	CDAI score mean (SD)
Sandborn 2014	Phase 2—Induction	4 weeks	Placebo		34	35.7 (12.7)	22	1	306.4 (62.6)
			Tofacitinib	1 mg BID	36	36.6 (12.2)	11	4	300.3 (76.7)
			Tofacitinib	5 mg BID	34	38.7 (10.2)	20	1	297.7 (63.7)
			Tofacitinib	15 mg BID	35	38.1 (11.7)	17	4	308 (50.8)
Panes 2017 (induction study)	Phase 2—Induction	8 weeks	Placebo		91	37.2 (11.7)	60	70	313 (67.14)
			Tofacitinib	5 mg BID	86	40.2 (11.5)	32	68	314 (53.06)
			Tofacitinib	10 mg BID	86	39.3 (13.7)	47	66	320 (61.66)
			Tofacitinib	15 mg BID	16	41.3 (14.3)	7	11	328 (76.66)
Panes 2017 (maintenance study)	Phase 2—Maintenance	26 weeks	Placebo		59	41.5 (12.8)	32	39	NR
			Tofacitinib	5 mg BID	60	38.1 (11.9)	30	48	NR
			Tofacitinib	10 mg BID	61	39.0 (13.1)	24	48	NR
Verimere 2017	Phase 2—Induction	10 weeks	Placebo		44	35.1 (11.8)	26	28	298.6 (56.8)
			Filgotinib	200 mg QD	130	37.4 (11.6)	71	73	291.3 (53.8)
Sandborn 2020	Phase 2—induction	12 or 16 weeks	Placebo		37	42.0 (11.292)	24	49	296.750 (60.931)
			Upadacitinib	3 mg BID	39	39.750 (10.949)	19	52	300.250 (61.733)
			Upadacitinib	6 mg BID	37	44.0 (12.704)	21	51	355.250 (86.809)
			Upadacitinib	12 mg BID	36	42.750 (12.060)	17	49	307.500 (52.498)
			Upadacitinib	24 mg BID	36	43.250 (10.641)	25	50	318.250 (93.171)
			Upadacitinib	24 mg QD	35	41.750 (10.223)	19	47	315.500 (45.172)
Sandborn 2020	Phase 2—Maintenance	36 weeks	Upadacitinib	3 mg BID	60	NR	NR	NR	NR
			Upadacitinib	6 mg BID	23	NR	NR	NR	NR
			Upadacitinib	12 mg BID	59	NR	NR	NR	NR
			Upadacitinib	24 mg QD	36	NR	NR	NR	NR
D'Haens 2022	Phase 2—Maintenance	96 months	Upadacitinib	15 mg QD	53	41.25 (9.93)	29	NR	295.75 (62.9)
			Upadacitinib	30 mg QD	31	39.75 (10.96)	17	NR	301.25 (41.63)
			Upadacitinib	30 mg QD dose‐escalated	23	44 (14)	10	NR	305 (50.81)
D'Haens 2023	Phase 2—Induction	24 Weeks	Filgotinib	200 mg QD	28	46 (16.3)	19	16	309 (55.7)
			Filgotinib	100 mg QD	32	42 (12.9)	23	8	297 (64.9)
			Placebo		18	45 (12.9)	9	6	300 (63.7)
Loftus 2023 U‐EXCEL	Phase 3—Induction	12 weeks	Placebo		176	39.3 ± 13.6	NR	NR	293.9 (85.4)
			Upadacitinib	45 mg QD	350	39.7 ± 13.7	NR	NR	292.4 (81.3)
Loftus 2023 U‐ENDURE	Phase 3—Maintenance	52 weeks	Placebo		165	38.1 ± 13.0	NR	NR	308.4 (82.3)
			Upadacitinib	15 mg QD	169	38.1 ± 13.5	NR	NR	300.8 (90.8)
			Upadacitinib	30 mg QD	168	37.0 ± 13.3	NR	NR	312.1 (75.4)
Loftus 2023 U‐EXCEED	Phase 3—Induction	12 weeks	Placebo		171	37.5 ± 12.1	NR	NR	308.1 (84.3)
			Upadacitinib	45 mg QD	324	38.4 ± 13.7	NR	NR	306.6 (89.4)
Reinisch 2024	Phase 2—Induction	24 weeks	Placebo		15	39 (11.8)	4	4	190 (57.9)
			Filgotinib	100 mg QD	25	41 (14)	10	12	194 (67.1)
			Filgotinib	200 mg QD	17	39 (11.2)	9	6	190 (62.4)
Verimere 2025 A	Phase 3—Induction	10 weeks	Placebo		237	38 (14)	130	112	320 (59.4)
			Filgotinib	100 mg QD	245	39 (14.1)	106	111	322 (55.5)
			Filgotinib	200 mg QD	222	39 (13.8)	110	101	322 (55.5)
Verimere 2025 B	Phase 3—Induction	10 weeks	Placebo		229	39 (12.5)	115	227	322 (57.5)
			Filgotinib	100 mg QD	228	42 (13.5)	127	227	321 (55.7)
			Filgotinib	200 mg QD	202	39 (14.2)	114	201	306 (54.0)
Verimere 2025	Phase 3—Maintenance	58 weeks	Placebo‐Placebo		145	NR	NR	NR	NR
			Filgotinib‐Placebo	100 mg QD	55	NR	NR	NR	NR
			Filgotinib‐Filgotinib	100 mg QD‐100 mg QD	104	NR	NR	NR	NR
			Filgotinib‐Placebo	200 mg	56	NR	NR	NR	NR
			Filgotinib‐Filgotinib	200 mg QD‐200 mg QD	118	NR	NR	NR	NR

### 3.3 Quality Assessment of the Included Studies

**FIGURE 2:**
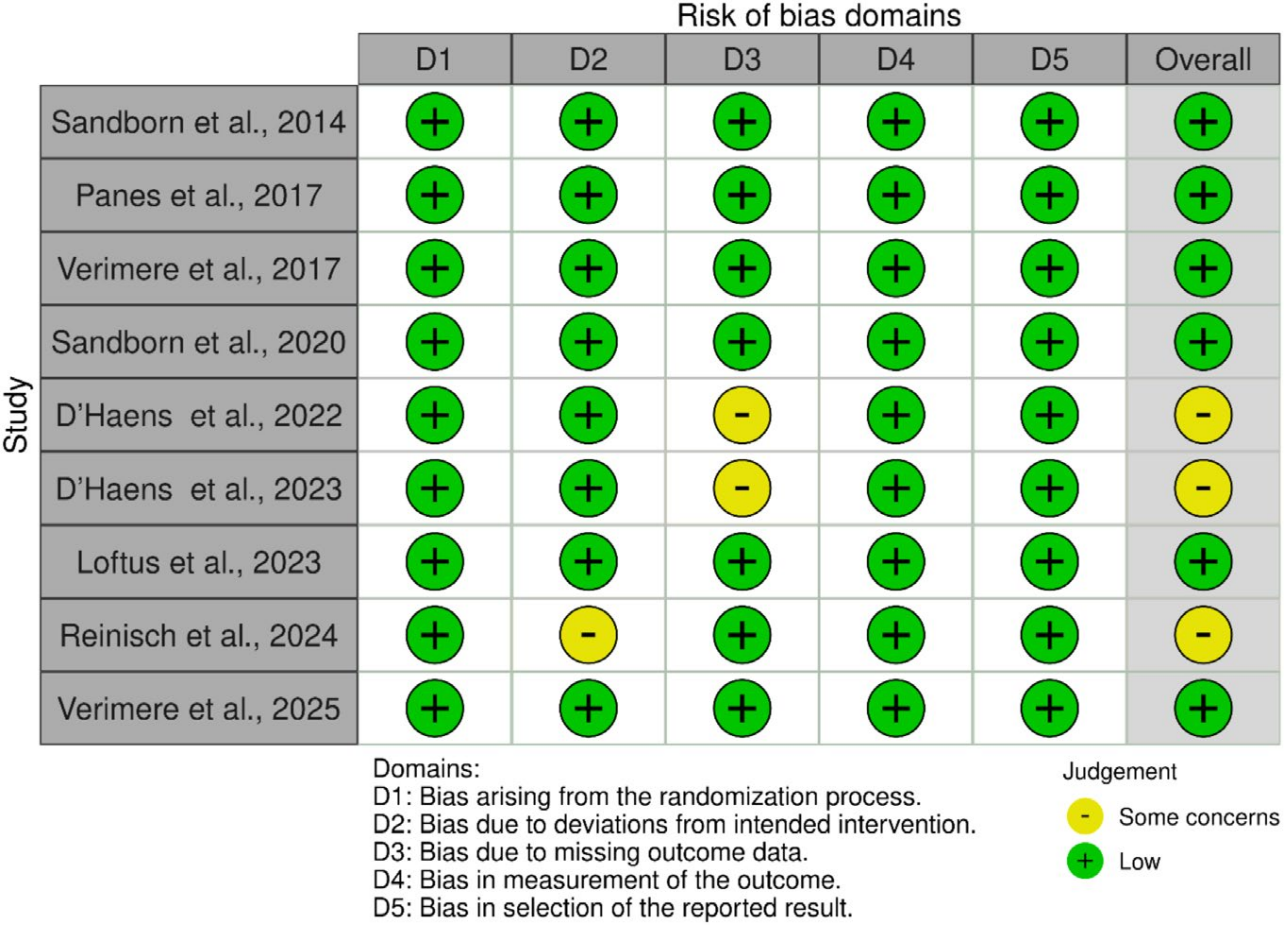
Risk of bias graph for RCTs (ROB2).

The quality of the included studies was evaluated using the Cochrane Risk of Bias (RoB‐2) [17], with the assessment results summarized in (Figure 2). Six studies demonstrated a low risk of bias across all assessed domains (CITE). Specifically, all studies had a low risk of bias in the randomization process. According to the bias due to deviations from intended interventions, it was consistently low across all studies except Reinisch et al. (2024). Regarding missing outcome data, seven studies were rated as low risk, while D'Haens et al. (2022) and D'Haens et al. (2023) presented some concerns due to incomplete outcome reporting. Measurement of outcomes was consistently low risk across all studies, ensuring reliable data collection methods. Additionally, all studies demonstrated a low risk of bias in the selection of the reported results, indicating transparent outcome reporting.

### 3.4 Clinical Efficacy Outcomes

Clinical efficacy was assessed using multiple endpoints to ensure that all aspects of clinical status are included, and to address the variability of reported outcomes across the included trials. CDAI remission was defined as achievement of a Crohn's Disease Activity Index score < 150, whereas Clinical Remission is a trial‐defined symptom‐dependent remission, and Clinical response‐100 represented a ≥ 100‐point reduction in CDAI from baseline.

#### 3.4.1 CDAI Remission

This section presents the clinical efficacy outcomes of 11 interventions, including various dosing regimens of filgotinib, tofacitinib, and upadacitinib, evaluated for CDAI‐based clinical remission in the network meta‐analysis (Table 2).

**TABLE 2:** Summary of efficacy outcomes for JAK inhibitors in Crohn's disease.

Treatment	CDAI remission (RR [95% CrI])	Clinical remission (RR [95% CrI])	CDAI mean difference (95% CrI)	Clinical response‐100 (RR [95% CrI])
Filgotinib_100_QD	1.0 (0.68, 1.7)			
Filgotinib_200_QD	1.4 (0.99, 2.2)			1.5 (0.82, 2.7)
Tofacitinib_1_BID	1.1 (0.54, 2.3)	1.7 (0.60, 4.6)	7.5 (−56, 74)	1.0 (0.45, 2.0)
Tofacitinib_5_BID	0.99 (0.48, 2.1)	1.2 (0.58, 2.5)	−2.6 (−57, 20)	1.3 (0.89, 2.1)
Tofacitinib_10_BID		1.2 (0.47, 2.8)	−3.8 (−78, 14)	1.3 (0.80, 2.2)
Tofacitinib_15_BID	1.1 (0.50, 2.2)	0.94 (0.40, 2.1)	−2.2 (−79, 37)	1.3 (0.80, 2.1)
Upadacitinib_3_BID	1.3 (0.40, 4.7)	1.2 (0.26, 5.6)		
Upadacitinib_6_BID	2.0 (0.66, 6.8)	2.7 (0.69, 11.)		
Upadacitinib_12_BID	2.5 (0.92, 8.4)	1.1 (0.20, 5.3)		
Upadacitinib_24_BID	2.0 (0.67, 6.8)	2.2 (0.56, 9.5)		
Upadacitinib_24_QD	1.3 (0.40, 4.6)	1.4 (0.30, 6.3)		
Upadacitinib_45_QD	1.8 (1.1, 3.0)			1.7 (1.2, 2.4)

**FIGURE 3:**
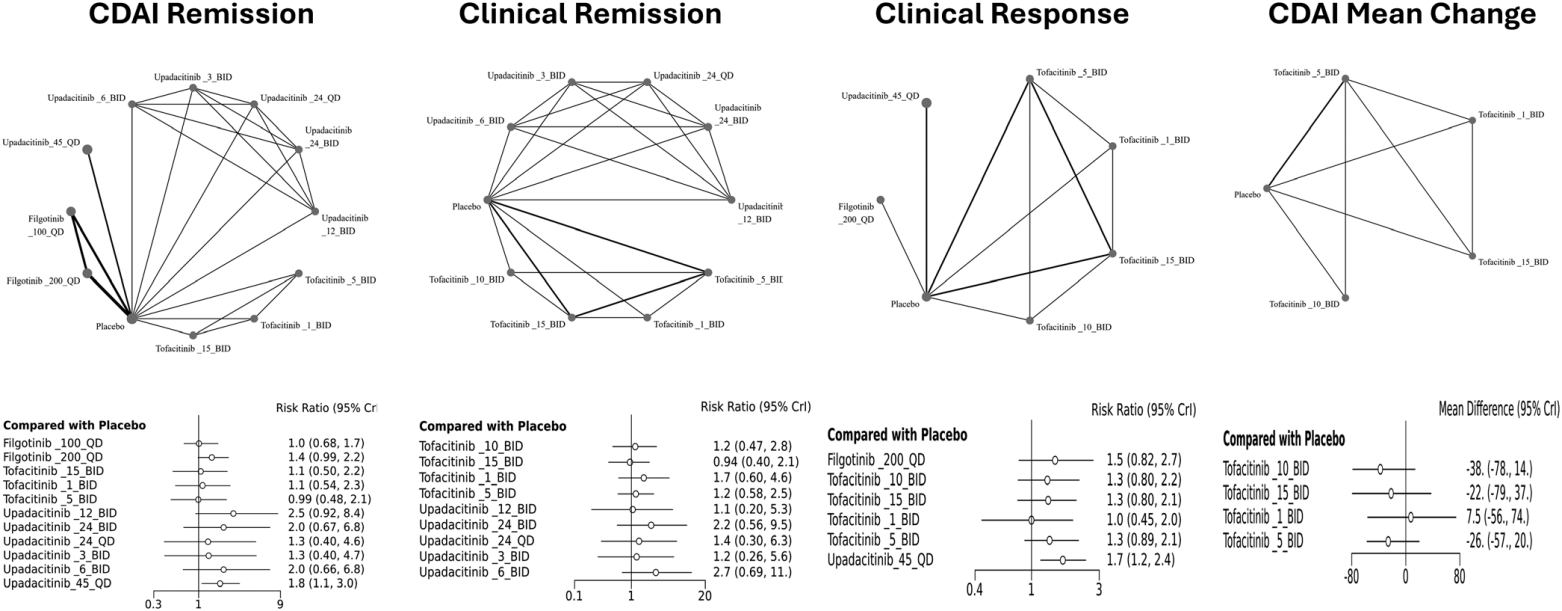
Network geometry of JAK inhibitors in Crohn's disease based on CDAI remission, clinical remission, clinical response, and CDAI mean change.

The network geometry (Figure 3) demonstrated a well‐connected structure, with placebo serving as the common comparator across all interventions. Direct comparisons were available for multiple doses of upadacitinib and tofacitinib, enhancing the reliability of the estimates. Among the evaluated treatments, upadacitinib 45 mg QD showed a statistically significant improvement in achieving CDAI remission compared to placebo (RR: 1.8, 95% CrI: 1.1–3.0). Other interventions, including filgotinib 200 mg QD (RR: 1.4, 95% CrI: 0.99–2.2), upadacitinib 12 mg BID (RR: 2.5, 95% CrI: 0.92–8.4), 6 mg BID (RR: 2.0, 95% CrI: 0.66–6.8), and 24 mg BID (RR: 2.0, 95% CrI: 0.67–6.8), demonstrated numerically higher remission rates compared to placebo. However, these differences were not statistically significant, as their 95% credible intervals included unity. These estimates are summarized in the league table presented in (Table 3).

**TABLE 3:** League table of risk ratios (RR) for JAK inhibitors and placebo in CDAI remission.

1.411 (0.898, 2.154)	0.991 (0.595, 1.478)	1.049 (0.418, 2.293)	1.105 (0.457, 2.442)	0.983 (0.398, 2.236)	2.493 (0.807, 8.584)	1.936 (0.588, 6.982)	1.268 (0.337, 4.657)	1.292 (0.355, 4.860)	1.937 (0.579, 6.842)	1.768 (0.843, 3.365)
Filgotinib _200_QD	0.702 (0.449, 1.015)	0.743 (0.301, 1.646)	0.782 (0.330, 1.727)	0.695 (0.292, 1.570)	1.769 (0.577, 6.010)	1.368 (0.437, 4.953)	0.896 (0.249, 3.395)	0.912 (0.256, 3.442)	1.361 (0.422, 4.948)	1.249 (0.626, 2.331)
	Placebo	1.059 (0.500, 2.158)	1.111 (0.536, 2.337)	0.991 (0.476, 2.115)	2.543 (0.924, 8.380)	1.957 (0.670, 6.777)	1.287 (0.397, 4.637)	1.310 (0.402, 4.696)	1.957 (0.660, 6.768)	1.780 (1.092, 2.998)
		Tofacitinib _15_BID	1.054 (0.534, 2.159)	0.936 (0.469, 2.014)	2.398 (0.712, 9.671)	1.866 (0.516, 7.562)	1.214 (0.293, 5.329)	1.240 (0.324, 5.353)	1.865 (0.514, 7.670)	1.686 (0.717, 4.253)
			Tofacitinib _1_BID	0.892 (0.433, 1.856)	2.268 (0.654, 8.952)	1.772 (0.497, 7.110)	1.147 (0.285, 4.896)	1.170 (0.305, 5.054)	1.748 (0.495, 6.954)	1.601 (0.662, 3.982)
				Tofacitinib _5_BID	2.538 (0.739, 10.325)	1.980 (0.541, 8.256)	1.281 (0.319, 5.773)	1.315 (0.336, 5.675)	1.967 (0.524, 7.979)	1.793 (0.749, 4.472)
					Upadacitinib _12_BID	0.782 (0.310, 1.919)	0.503 (0.176, 1.348)	0.515 (0.180, 1.334)	0.765 (0.302, 1.846)	0.707 (0.190, 2.224)
						Upadacitinib _24_BID	0.649 (0.217, 1.806)	0.663 (0.228, 1.837)	0.992 (0.382, 2.535)	0.908 (0.239, 2.950)
							Upadacitinib _24_QD	1.032 (0.330, 3.199)	1.526 (0.538, 4.524)	1.393 (0.359, 5.037)
								Upadacitinib _3_BID	1.475 (0.532, 4.365)	1.375 (0.338, 5.063)
									Upadacitinib _6_BID	0.910 (0.240, 3.009)
										Upadacitinib_45_QD

**FIGURE 4:**
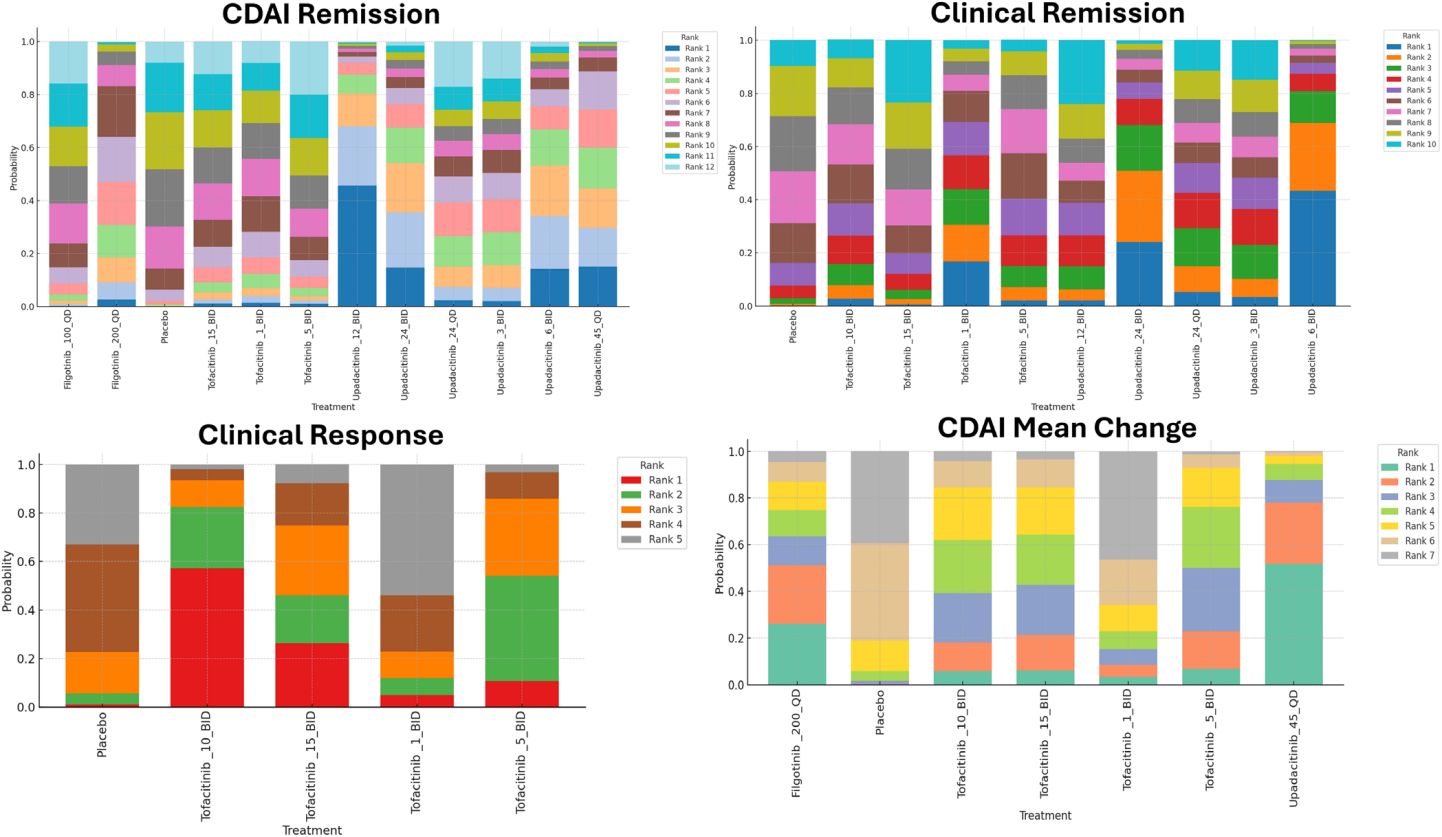
Surface Under the Cumulative Ranking curve (SUCRA) analysis of tofacitinib, filgotinib, and upadacitinib illustrating CDAI remission, clinical remission, clinical response, and CDAI mean change outcomes through rank probability bar charts.

**FIGURE 5:**
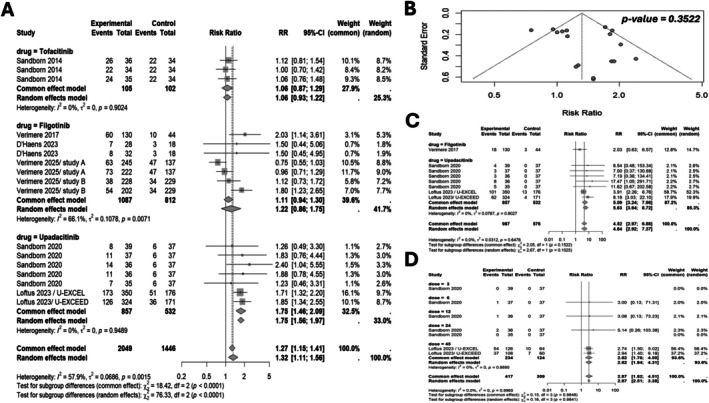
Forest plot and funnel plot analysis of risk ratios.

SUCRA analysis (Figure 4) indicated that upadacitinib 12 mg BID had the highest probability of being ranked the most effective intervention, followed by 45 mg QD and 6 mg BID. Conversely, placebo, filgotinib 100 mg QD, and lower tofacitinib doses consistently had lower SUCRA rankings, indicating a lower probability of achieving higher relative efficacy. Study‐level contributions (Figure 5) indicated that the largest proportion of remission events came from the U‐EXCEL and U‐EXCEED trials (upadacitinib 45 mg QD), while smaller studies like D'Haens 2023 and Sandborn 2014 contributed fewer events due to limited sample sizes (Table S1).

#### 3.4.2 Clinical Remission

This section presents the clinical remission outcomes from the network meta‐analysis (NMA) of 9 treatment regimens, including tofacitinib, upadacitinib, and filgotinib. Placebo served as the common comparator across all interventions (Table 2, Table S5).

Upadacitinib 6 mg BID showed the highest relative efficacy (RR: 2.7, 95% CrI: 0.69–11.0), followed by 24 mg BID (RR: 2.2, 95% CrI: 0.56–9.5), but these differences did not reach statistical significance (Figure 3). SUCRA analysis (Figure 4) ranked Upadacitinib 6 mg BID as the highest probability of being the most effective, followed by 24 mg BID and 45 mg QD.

Direct pairwise meta‐analysis results (Figure 5A) aligned with the network estimates, with Upadacitinib 45 mg QD showing the highest relative effect in most direct comparisons (RR: 1.75, 95% CI: 1.46–2.09), while filgotinib and tofacitinib exhibited lower effect sizes. Subgroup analysis (Figure 5D) revealed that higher doses of Upadacitinib (45 mg QD and 24 mg BID) were associated with greater numerical benefits. No significant publication bias was detected (Egger's *p* = 0.3522; Figure 5B). Direct event counts in Table S2.

#### 3.4.3 Clinical Response‐100

This section presents the Clinical Response‐100 outcomes for six active treatments, including tofacitinib, upadacitinib, and filgotinib, assessed in the network meta‐analysis (NMA) (Table 2).

Placebo served as the common comparator. Upadacitinib 45 mg QD emerged as the most efficacious intervention with a statistically significant improvement over placebo (RR: 1.7; 95% CrI: 1.2–2.4). Other interventions, including Filgotinib 200 mg QD, Tofacitinib 10 mg BID, 5 mg BID, and 15 mg BID, were associated with higher response rates than placebo. Tofacitinib 1 mg BID had an RR of 1.0 (95% CrI: 0.45–2.0), indicating no apparent benefit over placebo (Figure 3).

The SUCRA analysis for CDAI mean change demonstrated that Upadacitinib 45 QD had the highest probability of being the most effective treatment, followed by Filgotinib 200 QD. In contrast, Tofacitinib 1 BID and placebo showed the highest probabilities of being ranked among the least effective interventions. Overall, Upadacitinib and Filgotinib were associated with the most favorable efficacy rankings, while lower‐dose Tofacitinib regimens and placebo consistently ranked lower (Figure 4). The league table (Table S6) further detailed pairwise comparisons, with Upadacitinib 45 mg QD consistently showing higher response rates. Direct event counts (Table S3) further supported these findings, with Upadacitinib achieving higher response rates in key trials as U‐EXCEL and U‐EXCEED.

#### 3.4.4 Change in CDAI Score

The NMA results (Table 2) indicated that all active treatments achieved greater reductions in CDAI scores compared to placebo. Tofacitinib 10 mg BID showed the largest numerical reduction (MD: −38; 95% CrI: −78 to 14), followed by 5 mg BID (MD: −26; 95% CrI: −57 to 20).

Direct trial data (Table S4) generally supported these findings, with Tofacitinib 10 mg BID consistently showing the largest numerical improvement. However, even this regimen showed overlapping confidence intervals with placebo, indicating limited statistical reliability. The SUCRA analysis (Figure 4) showed that Tofacitinib 10 mg BID had the highest probability of being the most effective for Clinical Response‐100, followed by 15 mg BID. Results for the mean change in CDAI are further detailed in the league table (Table S7).

## 4 Dose–Response and Meta‐Regression Analysis

**FIGURE 6:**
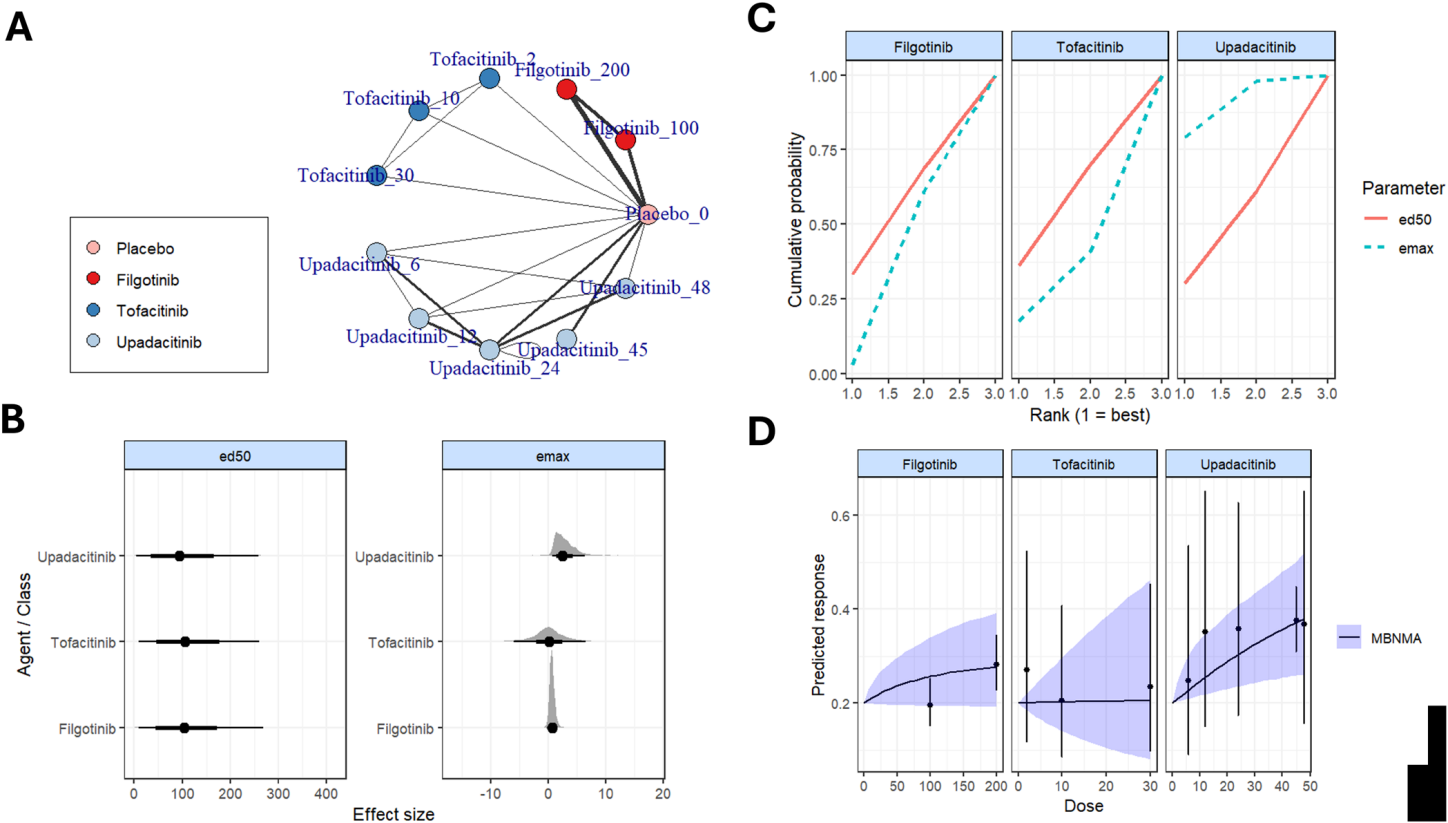
Dose–response and pharmacodynamic curves for each JAK inhibitor.

Dose–response modeling (Figure 6A–D) indicated a positive relationship between higher dosing and efficacy, especially for upadacitinib. Upadacitinib exhibited a higher maximum effect (Emax) and a lower effective dose (ED50) compared to filgotinib and tofacitinib (Figure 6B,D).

Meta‐regression analysis (Table 4) revealed that upadacitinib was significantly more effective than filgotinib for achieving CDAI remission (Estimate: 0.872; 95% CI: 0.31–1.43; *p* = 0.005). Higher dosing was a significant positive moderator of efficacy (Estimate: 0.0042; 95% CI: 0.0002–0.0082; *p* = 0.041), while dosing frequency (QD vs. BID) had no significant impact (*p* = 0.436). Cumulative probability curves (Figure 4) further supported the superior ranking of upadacitinib, particularly at higher doses, highlighting its dose‐dependent efficacy. Diagnostics for the direct meta‐analyses across all outcomes are provided in Tables S1–S4.

**TABLE 4:** Results of the multiple meta‐regression analysis for CDAI remission, showing estimates, standard errors (SE), *p*‐values, 95% confidence intervals (CI), and significance levels for different moderators (drugs, dose, and frequency).

Moderator	Estimate	SE	*p*	95% CI	Significance
Intercept	−0.313	0.342	0.378	[−1.06, 0.43]	
Drug: Tofacitinib	0.342	0.353	0.351	[−0.43, 1.11]	
Drug: Upadacitinib	0.872	0.257	0.005	[0.31, 1.43]	Significant (**)
Dose	0.0042	0.0018	0.041	[0.0002, 0.0082]	Significant(*)
Frequency (QD vs. BID)	−0.197	0.245	0.436	[−0.73, 0.34]	Not significant

*Note:* Reference Categories: drug (Fligotinib), Frequency (BID).

## 5 Safety and Adverse Events

**FIGURE 7:**
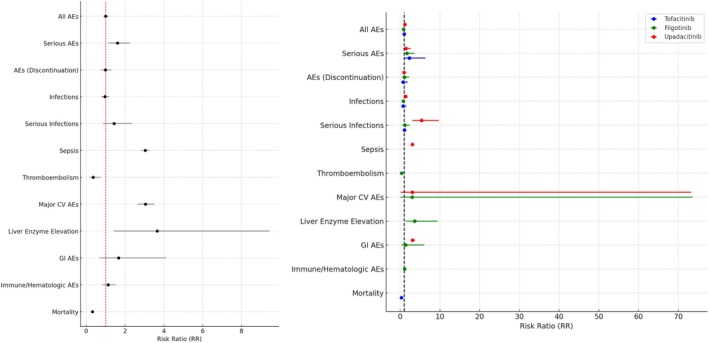
Safety and adverse events (AEs) of Janus Kinase (JAK) inhibitors in Crohn's disease.

The safety profiles of tofacitinib, filgotinib, and upadacitinib were evaluated across nine studies (Figure 7). Differences between fixed‐ and random‐effects models reflect substantial between‐study heterogeneity, with fixed‐effects estimates largely driven by larger trials, whereas random‐effects and stratified analyses better capture variability across agents and dosing regimens. Therefore, stratified analyses were prioritized to identify agent‐specific safety signals. Overall, treatment groups showed a significant reduction in adverse events (AEs) compared with placebo under a fixed‐effects model (RR: 0.86; 95% CI: 0.82–0.90); however, this effect was not sustained under the random‐effects model, reflecting high heterogeneity (*I* ^2^ = 90.4%).

Stratified analysis (Table S8) revealed that upadacitinib was associated with a higher risk of AEs (RR: 1.19; 95% CI: 1.07–1.33), serious infections (RR: 5.39; 95% CI: 2.98–9.74), and liver enzyme elevations (RR: 3.66; 95% CI: 1.42–9.44), raising safety concerns. In contrast, filgotinib demonstrated a favorable safety profile, with no signal of increased thromboembolic risk (RR: 0.36; 95% CI: 0.17–0.77) and no significant increase in infections. Tofacitinib showed a neutral profile, with no significant differences in AEs or infections but a lower mortality rate (RR: 0.32; 95% CI: 0.30–0.35).

The subgroup analysis (Table S9) confirmed significant between‐group differences in AEs and serious infections (*p* < 0.0001), highlighting the variability in safety profiles among the three agents. Overall, upadacitinib was associated with higher rates of adverse events, particularly serious infections, while filgotinib demonstrated the most favorable safety profile within the constraints of trial data.

## 6 Discussion

This NMA comprehensively assessed the comparative efficacy and safety of three JAKis, Upadacitinib, Filgotinib, and Tofacitinib. By synthesizing data from randomized controlled trials using both direct and indirect comparisons, we evaluated treatment efficacy across multiple clinically relevant outcomes. SUCRA probabilities were used to rank interventions based on their relative performance; however, these rankings should not be over interpreted, as they represent probability rankings rather than statistical certainty. Additionally, dose–response modeling provided insights into the relationship between drug dose and therapeutic effect. Evidence from these complementary analytical approaches indicates that Upadacitinib, particularly at higher doses, demonstrates the most consistent and robust clinical benefit. Although this is tempered by a comparatively higher incidence of adverse events.

According to CDAI‐based clinical remission, which is defined as a CDAI < 150, our analysis revealed that Upadacitinib 45 mg QD was the only regimen to demonstrate a statistically significant benefit over placebo. This finding aligns with results from the U‐EXCEL and U‐EXCEED trials, where Upadacitinib 45 mg QD consistently showed superior remission rates compared to placebo in both biologic‐naive and biologic‐experienced patients [22]. On the other hand, when SUCRA analysis was used to rank the probability of each treatment being the most effective for inducing CDAI remission, it showed that Upadacitinib 12 mg BID was most likely to be the most effective intervention, closely followed by Upadacitinib 45 mg QD. This discrepancy may be explained by the methodological differences between frequentist inference and probabilistic ranking.

Upadacitinib may have demonstrated superior efficacy in our results due to its unique properties compared to other JAK inhibitors. Upadacitinib demonstrates markedly higher selectivity for JAK1 over other Janus kinases in cellular assays, with > 40‐ and 130‐fold selectivity over JAK2 and JAK3, respectively, in engineered cell lines designed to isolate individual kinase activity [23]. Other studies report similar findings, noting approximately 60‐fold selectivity over JAK2 and more than 100‐fold over JAK3. This high degree of JAK1 selectivity supports its potent anti‐inflammatory efficacy [24].

Beyond its high selectivity, Upadacitinib also demonstrated superior pharmacodynamic properties, particularly in its dose–response behavior, exhibiting a higher Emax and a lower ED50 compared to Filgotinib and Tofacitinib. These properties may explain its consistent superiority in clinical outcomes across immune‐mediated diseases, as agents with lower ED50 values may allow effective induction of remission with reduced drug exposure, while higher Emax may be advantageous when maximal disease control is required. This efficacy extends to ulcerative colitis, where a 2022 network meta‐analysis identified Upadacitinib as the top‐ranking agent for inducing clinical response and remission within 2 weeks, outperforming most biologics and small molecules except tofacitinib [25]. Moreover, long‐term extension data in ulcerative colitis further support its durability, with over 70% of patients maintaining clinical remission through 96 weeks [26]. In patients with rheumatoid arthritis, a post hoc analysis showed that initial Upadacitinib resulted in earlier target achievement and longer time spent in low disease states [27]. Together, these findings underscore Upadacitinib's durable efficacy across multiple immune‐mediated conditions.

Our analysis revealed that higher total daily dosing of JAK inhibitors was a significant positive moderator of efficacy in achieving CDAI remission in Crohn's disease. This suggests that increasing the overall dose may enhance therapeutic response. In contrast, dosing frequency (QD vs. BID) did not significantly impact efficacy, indicating that the magnitude of JAK inhibition, rather than its temporal distribution, is the key determinant of clinical outcomes. This aligns with previous studies; for example, pharmacokinetic modeling on JAKis confirmed no added benefit of BID over QD dosing at the same total daily dose in patients with moderate‐to‐severe rheumatoid arthritis [28]. Similarly, exposure–response analysis of Upadacitinib demonstrated that once‐daily dosing regimens at matched total daily doses achieved equivalent efficacy to BID regimens [29].

Rates of serious adverse events, sepsis, major cardiovascular events, and hepatic enzyme elevations were all higher than with placebo, mirroring the class‐wide cardiovascular and mortality warnings issued by the FDA after the ORAL Surveillance study of tofacitinib but occurring here in Crohn's patients rather than rheumatoid arthritis [30, 31]. Venous thromboembolic events were infrequent in our pooled analysis and did not show a statistically significant increase; however, given limited follow‐up and low event rates in randomized trials, these findings should be interpreted cautiously. Several systematic reviews across different JAK inhibitors and patient populations have similarly reported no consistent increase in VTE risk compared with controls, even across varying dosing regimens [26, 27, 28, 29, 30, 31].

Subgroup analysis revealed significant differences in the risk of overall AEs among individual JAK inhibitors, an observation that echoes the heterogeneous safety signals seen across recent systematic assessments of JAK‐inhibitor use in IBD [32]. Upadacitinib carried the greatest burden of overall AEs, a pattern consistent with the higher incidence of non‐serious events reported throughout the phase III U‐ACHIEVE/U‐ACCOMPLISH program [33]. By contrast, Filgotinib displayed the most favorable tolerability profile compared to other JAKis, with the UK real‐world cohort also demonstrating good tolerance to Filgotinib [34].

When focusing on infections, Upadacitinib was associated with the highest frequency of infectious side effects, with a notable increase in serious infections. This finding aligns with studies that have specifically reported a higher frequency of infections associated with Upadacitinib, including serious infections. These studies reinforce the importance of close monitoring in patients receiving Upadacitinib, given the elevated infection risk observed in these patients [32, 35, 36]. In contrast, Filgotinib showed a modest reduction in infection risk, and Tofacitinib exhibited an infection profile comparable to that of placebo.

Despite the strength of the analysis, there are some limitations. The small sample sizes in some studies may affect the statistical precision of the results. Additionally, the relatively short follow‐up periods of the included trials limit our ability to assess the long‐term effects of these treatments. Future research should focus on larger studies with extended follow‐up to better understand the durability of treatment effects and long‐term safety profiles. Furthermore, because the included trials primarily enrolled patients with moderate‐to‐severe Crohn's disease and frequent prior biologic exposure, the generalizability of these findings to biologic‐naïve patients, those with mild disease, or long‐term maintenance beyond trial durations may be limited. Moreover, while this analysis provides insights into the efficacy of various dosing regimens, further studies exploring the optimal dosing schedules and their effects on different patient subgroups are needed. Investigating factors such as age, comorbidities, and genetic variations in response to treatment would be beneficial for personalizing Crohn's disease therapies.

## 7 Conclusion

This comprehensive network meta‐analysis provides an in‐depth evaluation of JAK inhibitors in the treatment of Crohn's disease, highlighting their substantial efficacy in achieving remission. Among the treatments assessed, Upadacitinib stands out as the most effective, particularly at higher doses, and demonstrates superior pharmacodynamic properties compared to other JAK inhibitors. However, despite these advantages, Upadacitinib carries more significant safety concerns, particularly with regard to serious infections. This study offers valuable insights into the comparative efficacy and safety of JAK inhibitors, supporting their role in Crohn's disease treatment. Further research is needed to better understand long‐term effects and optimize treatment strategies.

## Funding

The authors have nothing to report.

## Ethics Statement

Owing to the nature of this research design involving review of existing literature, it is exempt from review by the institutional review board (IRB). As a result, no ethical approval was required for this article.

## Consent

All authors have been offered the opportunity to read the manuscript and clearly declare and consent for publication after approval.

## Conflicts of Interest

The authors declare no conflicts of interest.

## Supporting information


**Table S1:** Summary of CDAI Remission Outcome.
**Table S2:** Summary of Clinical Remission Outcome.
**Table S3:** Summary of Clinical Response outcome.
**Table S4:** Summary of CDAI Mean Change.
**Table S5:** League Table for Clinical Remission Outcome.
**Table S6:** League Table for Clinical Response Outcome.
**Table S7:** League Table of Mean Differences in CDAI Score Between Treatments.
**Table S8:** Summary of Adverse Events Associated with JAK inhibitors.
**Table S9:** Summary of Adverse Events Associated with JAK inhibitors Stratified by Specific JAK Inhibitor.

**Data S1:** jgh370388‐sup‐0002‐Supplementaryfile2.docx.

**Data S2:** jgh370388‐sup‐0003‐Supplementaryfile3.pdf.

## Data Availability

All data generated and referenced in this study are included in this published article and the Supporting Information files.
