# Supplementary material for: JAK Inhibitors for Crohn's Disease: A Systematic Review and Dose–Response Network Meta‐Analysis of Efficacy and Safety
Source: JGH Open. 2026 Mar 13;10(3):e70388. doi: 10.1002/jgh3.70388 (PMC13097650; doi:10.1002/jgh3.70388)
Supplement: Supplementary file 1 — Table S1: Summary of CDAI Remission Outcome. Table S2: Summary of Clinical Remission Outcome. Table S3: Summary of Clinical Response outcome. Table S4: Summary of CDAI Mean Change. Table S5: League Table for Clinical Remission Outcome. Table S6: League Table for Clinical Response Outcome. Table S7: League Table of Mean Differences in CDAI Score Between Treatments. Table S8: Summary of Adverse Events Associated with JAK inhibitors. Table S9: Summary of Adverse Events Associated with JAK inhibitors Stratified by Specific JAK Inhibitor. [file JGH3-10-e70388-s003.docx]

**SUPPLEMENTARY TABLES:**

**Supplementary Table 1. Summary of CDAI Remission Outcome.**

| **Study** | **Treatment** | **Subjects with outcome** | **N** |
| --- | --- | --- | --- |
|  |  |  |  |
| D’Haens 2023 | Filgotinib _100_QD | 8 | 32 |
|  | Filgotinib _200_QD | 7 | 28 |
|  | Placebo | 3 | 18 |
| Verimere 2025/ study A | Filgotinib _100_QD | 63 | 245 |
|  | Filgotinib _200_QD | 73 | 222 |
|  | Placebo | 47 | 137 |
| Verimere 2025/ study B | Filgotinib _100_QD | 38 | 228 |
|  | Filgotinib _200_QD | 54 | 202 |
|  | Placebo | 34 | 229 |
| Verimere 2017 | Filgotinib _200_QD | 60 | 130 |
|  | Placebo | 10 | 44 |
| Sandborn 2014 | Placebo | 22 | 34 |
|  | Tofacitinib _15_BID | 24 | 35 |
|  | Tofacitinib _1_BID | 26 | 36 |
|  | Tofacitinib _5_BID | 22 | 34 |
| Sandborn 2020 | Placebo | 6 | 37 |
|  | Upadacitinib _12_BID | 14 | 36 |
|  | Upadacitinib _24_BID | 11 | 36 |
|  | Upadacitinib _24_QD | 7 | 35 |
|  | Upadacitinib _3_BID | 8 | 39 |
|  | Upadacitinib _6_BID | 11 | 37 |
| Loftus 2023 / U-EXCEL | Placebo | 51 | 176 |
|  | Upadacitinib_45_QD | 173 | 350 |
| Loftus 2023/ U-EXCEED | Placebo | 36 | 171 |
|  | Upadacitinib_45_QD | 126 | 324 |

**Supplementary Table 2.** **Summary of Clinical Remission Outcome.**

| **Study** | **Treatment** | **Subjects with outcome** | **N** |
| --- | --- | --- | --- |
|  |  |  |  |
| Sandborn 2014 | Placebo | 7 | 34 |
|  | Tofacitinib _15_BID | 5 | 35 |
|  | Tofacitinib _1_BID | 11 | 36 |
|  | Tofacitinib _5_BID | 8 | 34 |
| Panes 2017 | Placebo | 33 | 92 |
|  | Tofacitinib _10_BID | 37 | 86 |
|  | Tofacitinib _15_BID | 7 | 16 |
|  | Tofacitinib _5_BID | 37 | 86 |
| Sandborn 2020 | Placebo | 4 | 37 |
|  | Upadacitinib _12_BID | 4 | 36 |
|  | Upadacitinib _24_BID | 8 | 36 |
|  | Upadacitinib _24_QD | 5 | 35 |
|  | Upadacitinib _3_BID | 5 | 39 |
|  | Upadacitinib _6_BID | 10 | 37 |

**Supplementary table 3. Summary of Clinical Response outcome.**

| **Study** | **Treatment** | **Subjects with outcome** | **N** |
| --- | --- | --- | --- |
|  |  |  |  |
| Verimere 2017 | Filgotinib _200_QD | 76 | 130 |
|  | Placebo | 18 | 44 |
| Sandborn 2014 | Placebo | 10 | 34 |
|  | Tofacitinib _15_BID | 13 | 35 |
|  | Tofacitinib _1_BID | 11 | 36 |
|  | Tofacitinib _5_BID | 15 | 34 |
| Panes 2017 | Placebo | 49 | 92 |
|  | Tofacitinib _10_BID | 59 | 86 |
|  | Tofacitinib _15_BID | 12 | 16 |
|  | Tofacitinib _5_BID | 60 | 86 |
| Loftus 2023 / U-EXCEL | Placebo | 65 | 176 |
|  | Upadacitinib_45_QD | 198 | 350 |
| Loftus 2023/ U-EXCEED | Placebo | 47 | 171 |
|  | Upadacitinib_45_QD | 163 | 324 |

**Supplementary table 4. Summary of CDAI Mean Change.**

| **Study** | **Treatment** | **μ** | **σ** | **N** |
| --- | --- | --- | --- | --- |
|  |  |  |  |  |
| Sandborn 2014 | Placebo | -68.6 | 93.59 | 34 |
|  | Tofacitinib _15_BID | -78.5 | 94.45 | 35 |
|  | Tofacitinib _1_BID | -48.5 | 129.7 | 36 |
|  | Tofacitinib _5_BID | -64.3 | 112.3 | 34 |
| Panes 2017 | Placebo | -117 | 10.3 | 92 |
|  | Tofacitinib _10_BID | -157 | 10.7 | 86 |
|  | Tofacitinib _5_BID | -150 | 10.7 | 86 |

**Supplementary table 5. League Table for Clinical Remission Outcome.**

| **Clinical Remission** | |  |  |  |  |  |  |  |  |
| --- | --- | --- | --- | --- | --- | --- | --- | --- | --- |
| **Placebo** | 1.167 ( 0.470, 2.764) | 0.944 ( 0.397, 2.069) | 1.652 ( 0.596, 4.619) | 1.196 ( 0.580, 2.469) | 1.054 ( 0.199, 5.343) | 2.228 ( 0.562, 9.450) | 1.363 ( 0.295, 6.287) | 1.237 ( 0.258, 5.567) | 2.695 ( 0.690, 11.410) |
|  | **Tofacitinib _10_BID** | 0.819 ( 0.303, 2.023) | 1.421 ( 0.436, 4.866) | 1.023 ( 0.437, 2.419) | 0.911 ( 0.142, 5.795) | 1.904 ( 0.383, 10.252) | 1.157 ( 0.211, 7.103) | 1.073 ( 0.183, 6.262) | 2.287 ( 0.471, 12.584) |
|  |  | **Tofacitinib _15_BID** | 1.764 ( 0.621, 5.370) | 1.251 ( 0.589, 2.986) | 1.139 ( 0.175, 6.923) | 2.395 ( 0.471, 12.678) | 1.424 ( 0.261, 8.765) | 1.341 ( 0.234, 7.593) | 2.887 ( 0.574, 15.459) |
|  |  |  | **Tofacitinib _1_BID** | 0.719 ( 0.253, 1.985) | 0.649 ( 0.086, 4.141) | 1.373 ( 0.239, 7.707) | 0.817 ( 0.129, 5.182) | 0.754 ( 0.120, 4.607) | 1.618 ( 0.293, 8.978) |
|  |  |  |  | **Tofacitinib _5_BID** | 0.889 ( 0.144, 5.348) | 1.852 ( 0.389, 9.546) | 1.149 ( 0.215, 6.510) | 1.038 ( 0.183, 5.599) | 2.262 ( 0.475, 11.056) |
|  |  |  |  |  | **Upadacitinib _12_BID** | 2.096 ( 0.555, 9.816) | 1.300 ( 0.299, 6.422) | 1.182 ( 0.260, 5.793) | 2.505 ( 0.704, 10.953) |
|  |  |  |  |  |  | **Upadacitinib _24_BID** | 0.614 ( 0.155, 2.246) | 0.563 ( 0.140, 2.116) | 1.197 ( 0.371, 3.787) |
|  |  |  |  |  |  |  | **Upadacitinib _24_QD** | 0.921 ( 0.209, 3.770) | 1.946 ( 0.549, 7.456) |
|  |  |  |  |  |  |  |  | **Upadacitinib _3_BID** | 2.141 ( 0.605, 8.619) |
|  |  |  |  |  |  |  |  |  | **Upadacitinib _6_BID** |

**Supplementary table 6. League Table for Clinical Response Outcome.**

| **Clinical Response** | |  |  |  |  |  |
| --- | --- | --- | --- | --- | --- | --- |
| **Filgotinib _200_QD** | 0.682 ( 0.368, 1.226) | 0.884 ( 0.413, 1.938) | 0.889 ( 0.410, 1.864) | 0.676 ( 0.253, 1.651) | 0.917 ( 0.454, 1.937) | 1.136 ( 0.554, 2.238) |
|  | **Placebo** | 1.293 ( 0.803, 2.168) | 1.310 ( 0.798, 2.066) | 0.999 ( 0.446, 1.956) | 1.340 ( 0.895, 2.147) | 1.663 ( 1.159, 2.413) |
|  |  | **Tofacitinib _10_BID** | 1.011 ( 0.589, 1.637) | 0.768 ( 0.326, 1.604) | 1.038 ( 0.656, 1.695) | 1.284 ( 0.690, 2.281) |
|  |  |  | **Tofacitinib _15_BID** | 0.763 ( 0.356, 1.499) | 1.027 ( 0.680, 1.654) | 1.270 ( 0.716, 2.324) |
|  |  |  |  | **Tofacitinib _1_BID** | 1.345 ( 0.714, 2.951) | 1.675 ( 0.780, 3.976) |
|  |  |  |  |  | **Tofacitinib _5_BID** | 1.247 ( 0.693, 2.072) |
|  |  |  |  |  |  | **Upadacitinib_45_QD** |

**Supplementary table 7. League Table of Mean Differences in CDAI Score Between Treatments.**

| CDAI Mean Difference | | |  |  |
| --- | --- | --- | --- | --- |
| **Placebo** | -37.658 ( -78.488, 13.678) | -21.707 ( -79.195, 37.387) | 7.495 ( -56.372, 74.272) | -25.963 ( -57.438, 19.827) |
|  | **Tofacitinib _10_BID** | 14.054 ( -56.635, 81.168) | 43.120 ( -30.894, 116.651) | 10.096 ( -30.659, 59.590) |
|  |  | **Tofacitinib _15_BID** | 29.688 ( -37.505, 98.544) | -2.259 ( -58.376, 60.042) |
|  |  |  | **Tofacitinib _1_BID** | -31.258 ( -96.158, 36.400) |
|  |  |  |  | **Tofacitinib _5_BID** |

**Supplementary table 8. Summary of Adverse Events Associated with JAK inhibitors.**

| Adverse Event Type | k | Events | Common Effect RR (95% CI) | p | Random Effects RR (95% CI) | p | τ² | I² [95% CI] |
| --- | --- | --- | --- | --- | --- | --- | --- | --- |
| All AEs | 19 | 2119 | 0.8570 [0.8171–0.8988] | **<0.0001** | 1.0028 [0.8753–1.1488] | 0.9665 | 0.0707 | 90.4% [86.5%; 93.2%] |
| Serious AEs | 18 | 184 | 1.4339 [1.0542–1.9503] | **0.0217** | 1.6102 [1.1505–2.2537] | **0.0082** | 0.0991 | 0.0% [0.0%; 50.0%] |
| AEs (Discontinuation) | 16 | 279 | 1.0312 [0.8178–1.3003] | 0.7951 | 0.9922 [0.7535–1.3065] | 0.9525 | 0.0467 | 9.2% [0.0%; 46.0%] |
| Infections | 17 | 654 | 0.9200 [0.8069–1.0488] | 0.2123 | 0.9594 [0.7848–1.1729] | 0.6679 | 0.0785 | 46.4% [5.6%; 69.6%] |
| Serious Infections | 13 | 62 | 1.4314 [0.8326–2.4610] | 0.1945 | 1.4314 [0.8680–2.3606] | 0.1442 | 0 | 0.0% [0.0%; 56.6%] |
| Sepsis | 4 | 4 | 3.0430 [0.6242–14.8345] | 0.1685 | 3.0430 [2.8282–3.2742] | **<0.0001** | 0 | 0.0% [0.0%; 84.7%] |
| Thromboembolism | 2 | 2 | 0.3556 [0.0371–3.4059] | 0.3698 | 0.3556 [0.1651–0.7661] | **0.0371** | 0 | 0.00% |
| Major CV AEs | 2 | 2 | 3.0478 [0.3213–28.9084] | 0.3316 | 3.0478 [2.6391–3.5197] | **0.0065** | 0 | 0.00% |
| Liver Enzyme Elevation | 3 | 6 | 3.6596 [0.6365–21.0407] | 0.146 | 3.6596 [1.4180–9.4448] | **0.0277** | 0 | 0.0% [0.0%; 89.6%] |
| GI AEs | 6 | 15 | 1.6697 [0.5691–4.8984] | 0.3505 | 1.6697 [0.6754–4.1275] | 0.2052 | 0 | 0.0% [0.0%; 74.6%] |
| Immune/Hematologic AEs | 4 | 77 | 1.1273 [0.7227–1.7585] | 0.5972 | 1.1273 [0.8208–1.5483] | 0.3155 | 0 | 0.0% [0.0%; 84.7%] |
| Mortality | 3 | 3 | 0.3240 [0.0521–2.0159] | 0.2269 | 0.3240 [0.3021–0.3475] | **0.0002** | 0 | 0.0% [0.0%; 89.6%] |

**Supplementary table 9. Summary of Adverse Events Associated with JAK inhibitors Stratified by Specific JAK Inhibitor.**

| AE Type | Total k | Total Events | Tofacitinib RR [95% CI] | Filgotinib RR [95% CI] | Upadacitinib RR [95% CI] | Subgroup p-value |
| --- | --- | --- | --- | --- | --- | --- |
| All AEs | 19 | 2119 | 1.0079 [0.9434–1.0767] | 0.8321 [0.6159–1.1243] | 1.1927 [1.0675–1.3326] | **0.0008** |
| Serious AEs | 18 | 184 | 2.3298 [0.8532–6.3619] | 1.7322 [0.8389–3.5769] | 1.4620 [0.7945–2.6902] | 0.5109 |
| AEs (Discontinuation) | 16 | 279 | 0.7182 [0.2645–1.9502] | 1.0855 [0.5242–2.2481] | 0.9460 [0.5859–1.5275] | 0.624 |
| Infections | 17 | 654 | 0.7394 [0.3427–1.5951] | 0.8037 [0.5894–1.0958] | 1.3818 [1.1265–1.6950] | **<0.0001** |
| Serious Infections | 13 | 62 | 1.0698 [1.0698–1.0698]* | 1.1975 [0.5925–2.4203] | 5.3912 [2.9843–9.7395] | **<0.0001** |
| Sepsis | 4 | 4 | NA | NA | 3.0430 [2.8282–3.2742] | NA |
| Thromboembolism | 2 | 2 | NA | 0.3556 [0.1651–0.7661] | NA | NA |
| Major CV AEs | 2 | 2 | NA | 3.0131 [0.1234–73.5777] | 3.0822 [0.1297–73.2324] | 0.9921 |
| Liver Enzyme Elevation | 3 | 6 | NA | 3.6596 [1.4180–9.4448] | NA | NA |
| GI AEs | 6 | 15 | NA | 1.3833 [0.3136–6.1015] | 3.1253 [2.6197–3.7285] | 0.0806 |
| Immune/Hematologic AEs | 4 | 77 | NA | 1.1273 [0.8208–1.5483] | NA | NA |
| Mortality | 3 | 3 | 0.3240 [0.3021–0.3475] | NA | NA | NA |
