# Supplementary material for: JAK Inhibitors for Crohn's Disease: A Systematic Review and Dose–Response Network Meta‐Analysis of Efficacy and Safety
Source: JGH Open. 2026 Mar 13;10(3):e70388. doi: 10.1002/jgh3.70388 (PMC13097650; doi:10.1002/jgh3.70388)
Supplement: Supplementary file 2 — Data S1: jgh370388‐sup‐0002‐Supplementaryfile2.docx. [file JGH3-10-e70388-s001.docx]

**Supplementary Material**

*Bayesian Network Meta-Analysis*

**Content:**

- - Prior Distributions Utilized In Bayesian Network Meta-Analysis
  - Convergence Diagnostics
  - Model Fit Assessment

**Bayesian Network Meta-Analysis (gemtc)**

1. Outcome: CDAI

CDAI ( higher is better , log scale )

| model type | network |
| --- | --- |
| effects type | random |
| outcome scale | 0.8290147 (imputed) |
| likelihood / link | binom / log |
| heterogeneity prior | standard deviation ~ uniform (0, 0.8290147) |
| run-length | - burn-in: 5000 - inference: 20000 - thinning: 10 |

| **Per-parameter convergence diagnostics** | | | |  |  |
| --- | --- | --- | --- | --- | --- |
|  |  |  |  | | |
| **Parameter** | **Standard deviation** | **Time-series S.E.** | **Parameter** | **Standard deviation** | |
| d.3.1 (Placebo, Filgotinib _100_QD) | 0.2270514 | 0.002954 | d.3.1 (Placebo, Filgotinib _100_QD) | 0.2270514 | 0.002954 |
| d.3.2 (Placebo, Filgotinib _200_QD) | 0.20328 | 0.0024938 | d.3.2 (Placebo, Filgotinib _200_QD) | 0.20328 | 0.0024938 |
| d.3.4 (Placebo, Tofacitinib _15_BID) | 0.3592176 | 0.0051783 | d.3.4 (Placebo, Tofacitinib _15_BID) | 0.3592176 | 0.0051783 |
| d.3.5 (Placebo, Tofacitinib _1_BID) | 0.3494692 | 0.0048483 | d.3.5 (Placebo, Tofacitinib _1_BID) | 0.3494692 | 0.0048483 |
| d.3.6 (Placebo, Tofacitinib _5_BID) | 0.366711 | 0.0050703 | d.3.6 (Placebo, Tofacitinib _5_BID) | 0.366711 | 0.0050703 |
| d.3.7 (Placebo, Upadacitinib _12_BID) | 0.5521013 | 0.0188798 | d.3.7 (Placebo, Upadacitinib _12_BID) | 0.5521013 | 0.0188798 |
| d.3.8 (Placebo, Upadacitinib _24_BID) | 0.575799 | 0.0185102 | d.3.8 (Placebo, Upadacitinib _24_BID) | 0.575799 | 0.0185102 |
| d.3.9 (Placebo, Upadacitinib _24_QD) | 0.6265911 | 0.0200525 | d.3.9 (Placebo, Upadacitinib _24_QD) | 0.6265911 | 0.0200525 |
| d.3.10 (Placebo, Upadacitinib _3_BID) | 0.6137365 | 0.0193315 | d.3.10 (Placebo, Upadacitinib _3_BID) | 0.6137365 | 0.0193315 |
| d.3.11 (Placebo, Upadacitinib _6_BID) | 0.5780165 | 0.0185515 | d.3.11 (Placebo, Upadacitinib _6_BID) | 0.5780165 | 0.0185515 |
| d.3.12 (Placebo, Upadacitinib_45_QD) | 0.2445687 | 0.0030043 | d.3.12 (Placebo, Upadacitinib_45_QD) | 0.2445687 | 0.0030043 |
| sd.d (Random effects standard deviation) | 0.1644408 | 0.0039308 | sd.d (Random effects standard deviation) | 0.1644408 | 0.0039308 |

| **Model fit statistics** | |
| --- | --- |
| Residual deviance ( D_res_) | 25.0 |
| Leverage (p_D_) | 22.3 |
| DIC | 47.4 |
| Number of data points | 25 |

1. Outcome: Clinical Remission / Induction Trials

| model type | network |
| --- | --- |
| effects type | **random** |
| outcome scale | **0.8472979 (imputed)** |
| likelihood / link | **binom / log** |
| heterogeneity prior | **standard deviation ~ uniform (0, 0.8472979)** |
| run-length | - **burn-in: 5000** - **inference: 20000** - **thinning: 10** |

| **Per-parameter convergence diagnostics** | | | |  |  |
| --- | --- | --- | --- | --- | --- |
|  |  |  | **Potential scale reduction factor (PSRF)** | | |
| **Parameter** | **Standard deviation** | **Time-series S.E.** | **Point estimate** | **97.5% quantile** | |
| d.1.2 (Placebo, Tofacitinib _10_BID) | 0.4182216 | 0.0054461 | 0.9998633 | 1.000341 |  |
| d.1.3 (Placebo, Tofacitinib _15_BID) | 0.4163236 | 0.0073684 | 1.00233 | 1.005402 |  |
| d.1.4 (Placebo, Tofacitinib _1_BID) | 0.5168866 | 0.0082104 | 1.007751 | 1.02438 |  |
| d.1.5 (Placebo, Tofacitinib _5_BID) | 0.3537599 | 0.0052888 | 1.000839 | 1.003343 |  |
| d.1.6 (Placebo, Upadacitinib _12_BID) | 0.8247321 | 0.0264447 | 1.002724 | 1.009238 |  |
| d.1.7 (Placebo, Upadacitinib _24_BID) | 0.7148039 | 0.0216351 | 1.003377 | 1.011105 |  |
| d.1.8 (Placebo, Upadacitinib _24_QD) | 0.7820357 | 0.0244591 | 1.004446 | 1.013066 |  |
| d.1.9 (Placebo, Upadacitinib _3_BID) | 0.7798241 | 0.0243891 | 1.002203 | 1.006357 |  |
| d.1.10 (Placebo, Upadacitinib _6_BID) | 0.7013607 | 0.0225871 | 1.005798 | 1.017078 |  |
| sd.d (Random effects standard deviation) | 0.2254444 | 0.0059717 | 1.001791 | 1.005881 |  |

| **Model fit statistics** | |
| --- | --- |
| Residual deviance ( D_res_) | 13.6 |
| Leverage (p_D_) | 13.0 |
| DIC | 26.6 |
| Number of data points | 14 |

1. CDAI Mean Change / Induction Trials

| model type | network |
| --- | --- |
| effects type | **random** |
| outcome scale | **39.9 (imputed)** |
| likelihood / link | **normal / identity** |
| heterogeneity prior | **standard deviation ~ uniform (0, 39.9)** |
| run-length | - **burn-in: 5000** - **inference: 20000** - **thinning: 10** |

| Per-parameter convergence diagnostics | |  |  |  |  |
| --- | --- | --- | --- | --- | --- |
|  |  |  | **Potential scale reduction factor (PSRF)** | | |
| **Parameter** | **Standard deviation** | **Time-series S.E.** | **Point estimate** | **97.5% quantile** | |
| d.1.2 (Placebo, Tofacitinib _10_BID) | 21.34978 | 0.2373753 | 1.001322 | 1.00424 |  |
| d.1.3 (Placebo, Tofacitinib _15_BID) | 28.86939 | 0.3627243 | 1.00052 | 1.00154 |  |
| d.1.4 (Placebo, Tofacitinib _1_BID) | 32.31461 | 0.4413947 | 0.999966 | 1.00056 |  |
| d.1.5 (Placebo, Tofacitinib _5_BID) | 18.54354 | 0.2339516 | 1.000089 | 1.0006 |  |
| sd.d (Random effects standard deviation) | 11.26911 | 0.2056271 | 1.004287 | 1.01369 |  |

| **Model fit statistics** | |
| --- | --- |
| Residual deviance ( D_res_) | 7.1 |
| Leverage (p_D_) | 6.6 |
| DIC | 13.7 |
| Number of data points | 7 |

1. Clinical Reponse-100 / Induction Trials

| model type | network |
| --- | --- |
| effects type | **random** |
| outcome scale | **0.5997526 (imputed)** |
| likelihood / link | **binom / log** |
| heterogeneity prior | **standard deviation ~ uniform (0, 0.5997526)** |
| run-length | - **burn-in: 5000** - **inference: 20000** - **thinning: 10** |

| **Per-parameter convergence diagnostics** | | | |  |  |
| --- | --- | --- | --- | --- | --- |
|  |  |  | **Potential scale reduction factor (PSRF)** | | |
| **Parameter** | **Standard deviation** | **Time-series S.E.** | **Point estimate** | **97.5% quantile** | |
| d.2.1 (Placebo, Filgotinib _200_QD) | 0.2954451 | 0.0039896 | 1.000185 | 1.000589 |  |
| d.2.3 (Placebo, Tofacitinib _10_BID) | 0.2404597 | 0.0033289 | 1.000714 | 1.001412 |  |
| d.2.4 (Placebo, Tofacitinib _15_BID) | 0.2359193 | 0.0042566 | 1.002958 | 1.008959 |  |
| d.2.5 (Placebo, Tofacitinib _1_BID) | 0.3722672 | 0.0079727 | 1.002014 | 1.004858 |  |
| d.2.6 (Placebo, Tofacitinib _5_BID) | 0.2127544 | 0.0034142 | 1.001178 | 1.00388 |  |
| d.2.7 (Placebo, Upadacitinib_45_QD) | 0.1724953 | 0.0023661 | 1.001125 | 1.003554 |  |
| sd.d (Random effects standard deviation) | 0.1367346 | 0.0032331 | 0.9999929 | 1.000424 |  |

| **Model fit statistics** | |
| --- | --- |
| Residual deviance ( D_res_) | 12.8 |
| Leverage (p_D_) | 12.0 |
| DIC | 24.9 |
| Number of data points | 14 |
