# Supplementary material for: JAK Inhibitors for Crohn's Disease: A Systematic Review and Dose–Response Network Meta‐Analysis of Efficacy and Safety
Source: JGH Open. 2026 Mar 13;10(3):e70388. doi: 10.1002/jgh3.70388 (PMC13097650; doi:10.1002/jgh3.70388)
Supplement: Supplementary file 3 — Data S2: jgh370388‐sup‐0003‐Supplementaryfile3.pdf. [file JGH3-10-e70388-s002.pdf]

Supplementary Material: Dose–Response Network Meta-Analysis

Network Description

A dose–response network meta-analysis (DR-NMA) was conducted to evaluate the comparative efficacy of Janus kinase (JAK) inhibitors for achieving CDAI remission in induction trials. The treatment network consisted of 8 randomized controlled trials, including 11 treatment nodes and 4 pharmacological agents (filgotinib, tofacitinib, upadacitinib, and placebo).

For each agent, a range of doses was evaluated, with a median of 4 doses per agent (range: 3–6). Both the agent-level and treatment-level networks were fully connected, allowing estimation of relative effects across all agents and doses through direct and indirect evidence synthesis.

Model Specification

A Bayesian model-based network meta-analysis (MBNMA) was implemented using a maximum effect ($\mathrm{E}_{\max}$) dose–response model. For binary outcomes (CDAI remission), a binomial likelihood with a logit link function was used.

Let p_{ik} denote the probability of remission in arm k of study i. The model was specified as:

$$\text{logit}(p_{ik}) = \begin{cases} \mu_i, & k = 1 \\ \mu_i + \delta_{ik}, & k \geq 2 \end{cases}$$

where μ_i represents the baseline (control arm) effect, and δ_{ik} is the relative treatment effect.

Relative effects were modeled as differences between dose–response functions:

$$\delta_{ik} = f(x_{ik}, t_{ik}) - f(x_{i1}, t_{i1})$$

The dose–response relationship was specified using an $\mathrm{E}_{\max}$ model:

$$f(x,t) = \frac{E_{\max,t} \cdot x}{ED_{50,t} + x}$$

where $E_{\max,t}$ denotes the maximum achievable treatment effect for agent t, and $ED_{50,t}$ is the dose at which 50% of the maximum effect is attained. The ED_{50} parameter was modeled on the log scale to ensure positivity.

Hierarchical Structure and Heterogeneity

To improve parameter identifiability and borrowing of information across agents, agent-specific $\mathrm{E}_{\max}$ and ED_{50} parameters were modeled hierarchically, assuming exchangeability across agents.

Between-study heterogeneity in relative treatment effects was modeled using a random-effects structure with a common between-study variance parameter.

Model Estimation

Models were estimated using Markov chain Monte Carlo (MCMC) sampling implemented via JAGS, interfaced through the `MBNMA` package in R. Three independent chains were run, each with sufficient burn-in and sampling iterations to ensure convergence. Convergence was assessed using trace plots and Gelman–Rubin diagnostics.

Parameter Estimates

Posterior distributions for the $\mathrm{E}_{\max}$ and ED_{50} parameters were derived. The estimated ED_{50} values differed across agents, indicating variation in dose potency, while $E_{\max}$ estimates reflected differences in maximal achievable efficacy. Credible intervals illustrate the uncertainty in parameter estimation, particularly for agents with fewer evaluated dose levels.

Treatment Ranking

Agent rankings were derived separately for the $E_{\max}$ and ED_{50} parameters using posterior rank probabilities. Ranking based on ED_{50} reflects relative potency (lower ED_{50} indicating higher potency), whereas ranking based on $E_{\max}$ reflects maximal achievable efficacy. Differences between ranking distributions highlight that agents with higher potency do not necessarily achieve the highest maximum effect.

Dose–Response Predictions

Posterior predictions of remission probability as a function of dose were generated for each agent. Predicted dose–response curves demonstrate increasing remission probability with dose, with plateauing effects consistent with an $\mathrm{E}_{\max}$ relationship. Shaded regions represent 95% credible intervals, reflecting uncertainty in predicted efficacy across the dose range. Observed trial-level data points are overlaid to illustrate model fit.

Interpretation

The $\mathrm{E}_{\max}$ MBNMA framework allowed coherent synthesis of dose–response information across agents while preserving within-trial randomization. The model supports comparative inference across both agents and doses, facilitates prediction at clinically relevant dose levels, and provides a principled basis for ranking treatments by potency and maximal efficacy.

Software

All analyses were conducted in R using the `MBNMA` package. Bayesian estimation was performed using JAGS.

Citation

Mawdsley, D., Bennetts, M., Dias, S., Boucher, M., Welton, N. (2016). ModelBased Network MetaAnalysis: a framework for evidence synthesis of clinical trial data. CPT Pharmacometrics Systems Pharmacology, 5(8), 393–401. https://doi.org/10.1002/psp4.12091
